# Exploring the therapeutic mechanism of curcumin in spinal cord injury treatment based on network pharmacology, molecular dynamics simulation, and experimental validation

**DOI:** 10.3389/fchem.2025.1568551

**Published:** 2025-03-28

**Authors:** Yongzhi He, Jiachun Lu, Yushan Luo, Rizhao Pang, Xiaoming Hu, Lijuan Ding, Hua Xiao, Yunyun Wang, Wenchun Wang

**Affiliations:** ^1^ School of Clinical Medicine, North Sichuan Medical College, Nanchong, Sichuan, China; ^2^ Department of Rehabilitation Medicine, General Hospital of Western Theater Command, Chengdu, Sichuan, China; ^3^ Department of Rehabilitation Medicine, Xichong County People’s Hospital, Nanchong, Sichuan, China; ^4^ Chengdu Eighth People’s Hospital (Geriatric Hospital of Chengdu Medical College), Chengdu, Sichuan, China; ^5^ College of Medicine, Southwest Jiaotong University, Chengdu, Sichuan, China; ^6^ The Second Affiliated Hospital of Guangzhou University of Chinese Medicine (Guangdong Provincial Hospital of Chinese Medicine), Guangzhou, Guangdong, China

**Keywords:** spinal cord injury, curcumin, natural products of plant origin, molecular mechanism, network pharmacology, molecular dynamics simulation, computer simulation

## Abstract

**Introduction:**

Curcumin, a natural active compound derived from plants, is widely used as a pigment across the globe. Research has demonstrated that curcumin possesses neuroprotective properties in spinal cord injuries (SCIs); however, its specific mechanisms of action remain unclear. This study aimed to elucidate the potential mechanisms underlying curcumin’s therapeutic effects in SCI.

**Methods:**

We screened the targets of curcumin in the treatment of spinal cord injury using network pharmacology across a variety of public databases. The interaction between the compound and the target was analyzed through bioinformatics analysis, molecular docking, and molecular dynamics simulation. Finally, the prediction results were verified by simulating spinal cord injury through oxygen–glucose deprivation (OGD) injury in PC12 cells.

**Results:**

Initial screening indicated 13 core targets involved in mitigating SCI. Curcumin may regulate the HIF pathway, immune cells, inflammation, oxidative stress, and other processes. Matrix metalloproteinase-9 (MMP9), tumor necrosis factor (TNF), interleukin-1β (IL-1β), signal transducer and activator of transcription 3 (STAT3), and caspase 3 (CASP3) were identified as key targets of curcumin in SCI regulation. Molecular docking results demonstrated that curcumin exhibited favorable affinity with the core targets, with MMP9 showing the highest binding affinity (−8.76 kcal/mol). Further studies confirmed that curcumin stably binds with MMP9, and the binding site was located at residues 220–225. Cell counting kit-8 (CCK8) assay results showed that curcumin exerted a good therapeutic effect. Western blot results showed that curcumin inhibited the expression of MMP9 protein but had no significant effect on the expression of TNF-α.

**Conclusion:**

Curcumin exerts its effects on SCI through multiple targets and pathways. Its specific mechanisms involve the inhibition of inflammation, prevention of apoptosis and ferroptosis, and promotion of neuronal repair. MMP9 may be a key target mediating curcumin’s protective effects against SCI. These findings provide scientific evidence for further research and development of drugs.

## Introduction

Spinal cord injury (SCI) represents a significant global burden with high morbidity and mortality, resulting in severe motor, sensory, and autonomic nerve impairments (Anjum et al., 2020). Over the past 30 years, its global prevalence has increased from 236 to 1,298 cases per million people. The global annual incidence of SCI is estimated to range between 250,000 and 500,000 individuals (Khorasanizadeh et al., 2019). Substantial resources are invested in gaining deeper insights into the pathophysiology of spinal cord injury and developing targeted therapies to enhance SCI outcomes; however, clinical translation remains limited. Currently, methylprednisolone and corticosteroids remain the primary pharmacological treatments; however, these therapies are associated with serious side effects such as wound infections, gastrointestinal bleeding, sepsis, pulmonary embolism, and increased mortality risk (Karsy and Hawryluk, 2019). Hence, discovering novel and effective therapeutic drugs holds significant research importance.

Curcumin is a natural pigment and food additive used worldwide. It is a polyphenolic metabolite derived from the root stems of turmeric (Mehla et al., 2020) and exhibits significant antioxidant, anti-inflammatory, and pharmacological effects. Its therapeutic efficacy in conditions such as traumatic brain injury, osteoarthritis, and other diseases has garnered considerable attention (Yang et al., 2023; Kahkhaie et al., 2019). Several studies have indicated that curcumin exerts a neuroprotective effect in spinal cord injury (Jiang et al., 2023; Jin et al., 2021), thus positioning it as a promising therapeutic agent. Nevertheless, the specific targets and mechanisms underlying curcumin’s treatment of spinal cord injury remain unclear.

**FIGURE 1:**
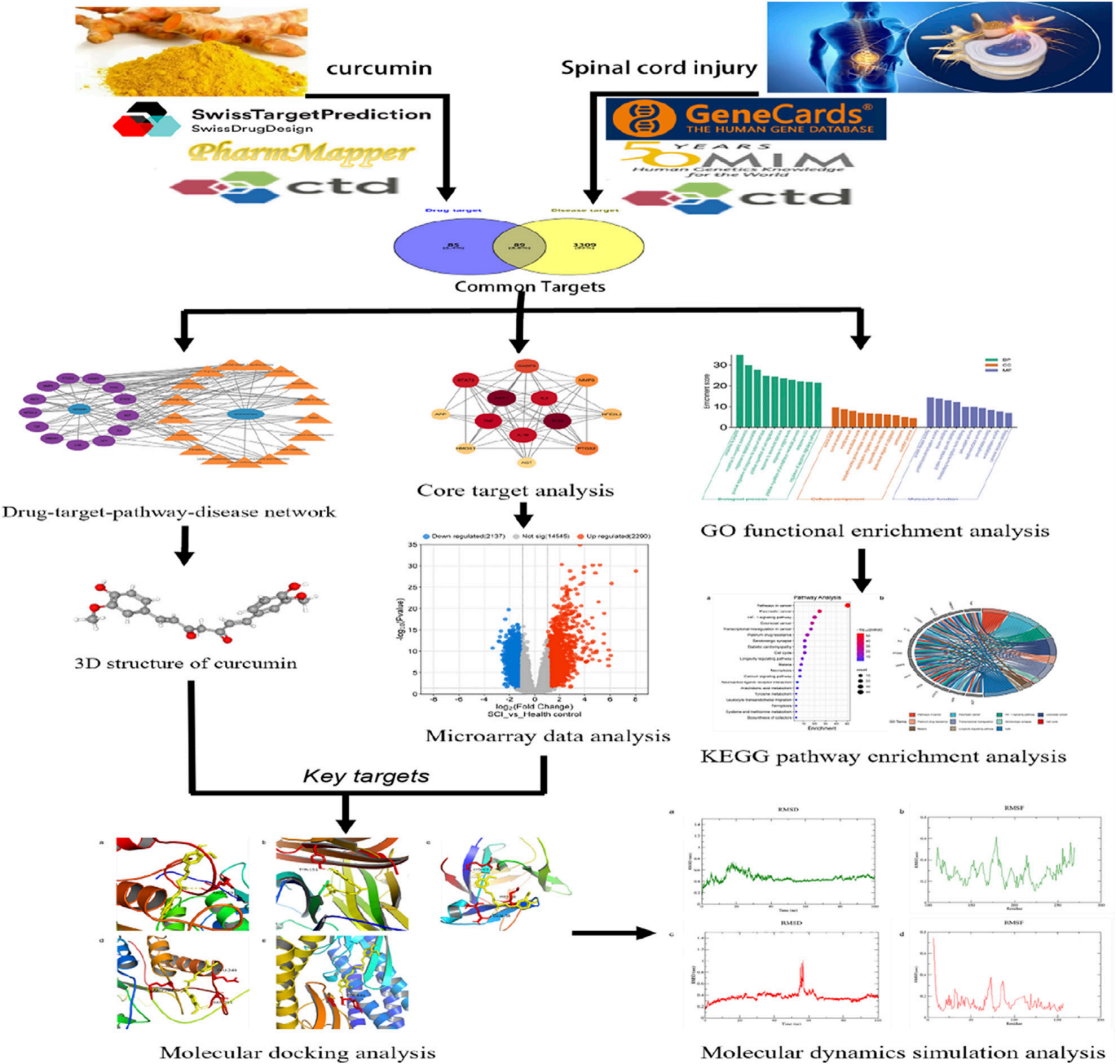
Workflow of investigating the potential mechanism of curcumin against spinal cord injury.

Emerging pharmacological research methodologies, such as molecular docking and molecular dynamics simulations, which integrate traditional pharmacological knowledge with bioinformatics techniques, can be employed to analyze potential drug targets, elucidate therapeutic effects and mechanisms in various diseases, and screen for suitable active ingredients in traditional Chinese medicine (Li Z. et al., 2023; Zhang P. et al., 2023). Through network analysis, microarray data analysis, molecular docking, and molecular dynamics simulation, this study comprehensively and systematically investigated the potential targets and complex mechanisms of curcumin’s therapeutic effects and verified the binding affinity and stability of curcumin to key targets, enriching its pharmacological mechanism of action and strengthening the scientific basis for future pharmacological research, drug development, and clinical translation (Figure 1).

## Materials and methods

### Screening of potential therapeutic targets of curcumin

The two-dimensional (2D) and three-dimensional (3D) chemical structures of curcumin were retrieved from PubChem (http://www.ncbi.nlm.nih.gov/home/chemicals) (Kim et al., 2016).The potential therapeutic targets of curcumin were identified using the SwissTargetPrediction Database (http://www.swisstargetprediction.ch/) (Daina et al., 2019), PharmMapper database (https://www.lilab-ecust.cn/pharmmapper/)(X. Wang et al., 2017), and Comparative Toxicogenomics Database (CTD) (https://ctdbase.ord/) (Davis et al., 2023).The SwissTargetPrediction database was employed to predict protein targets and functions for curcumin, using the SMILES number from PubChem (https://pubchem.ncbi.nlm.nih.gov/). The filtering criteria were set to include only human species and probabilities greater than 0. PharmMapper is a platform that integrates pharmacophore databases to identify potential targets using various algorithms. The SDF data of curcumin were uploaded, with a maximum of 300 pharmacophore models selected, and predicted protein targets with a Norm fit score >0.5 were used as the screening criterion.

Curcumin targets were searched in the CTD database, selecting those with an interaction count >20 for effective targeting. Redundant results from the three databases were removed and combined to identify potential therapeutic targets of curcumin.

### Screening of potential disease targets

Potential targets associated with spinal cord injury were identified through searches on GeneCards (https://www.genecards.org/) (Safran et al., 2010), the CTD (https://ctdbase.org/), and the Online Mendelian Inheritance in Man (OMIM) database (https://www.omim.org) (Amberger et al., 2015). The combined results from the three databases were deduplicated to identify potential targets related to spinal cord injury. Two groups of target intersections were considered to obtain curcumin together with spinal cord injury potential targets, using VENNY2.1 (https://bioinfogp.cnb.csic.es/tools/venny/) for visualization.

### PPI network construction and core target selection

The interaction target genes of curcumin and spinal cord injury were integrated into the STRING platform (http://string-db.org/) (Szklarczyk et al., 2023), with the organism set to *Homo sapiens* and the highest confidence threshold set to 0.7 as the selection condition, and a protein–protein interaction (PPI) network diagram constructed to visualize the interaction relationships between target proteins. Based on the exported data from STRING, core targets were selected using degree, closeness centrality, and betweenness centrality indicators in Cytoscape 3.10.0 software (Shannon et al., 2003) to generate a network diagram of the interactions between core targets (Sun et al., 2023).

### GO and KEGG enrichment analyses

The potential targets of curcumin against spinal cord injury were imported into the Metascape platform (http://metascape.org/) (Zhou et al., 2019) for Gene Ontology (GO) and Kyoto Encyclopedia of Genes and Genomes (KEGG) pathway enrichment analyses. A personalized analysis was conducted with “*Homo sapiens*” set as the target organism, and the selection criteria included minimum overlap of 3, a p-value threshold of 0.05, and a minimum concentration of 1.5 (Jiao Y. et al., 2023). Data from biological process (BP), cellular component (CC), molecular function (MF), and KEGG enrichment analyses were collected. Bioinformatics (https://www.bioinformatics.com.cn) (Tang et al., 2023) is used to generate GO bubble charts and KEGG pathway charts based on the results.

### Drug–disease–target–pathway network construction

To systematically analyze the potential molecular mechanism of curcumin against spinal cord injury, the core targets and the top 20 KEGG-enriched pathways were imported into Cytoscape 3.10.0 to construct the drug–disease–target–pathway network.

### Microarray data analysis

Further analysis was conducted on differentially expressed core target genes by downloading two datasets, namely, GSE151371 and GSE226238, from the Gene Expression Omnibus (GEO) database (https://www.ncbi.nlm.nih.gov/geo/) (Barrett et al., 2013). The GSE151371 dataset comprised data from 10 healthy controls and 38 patients with spinal cord injury, while the GSE226238 dataset included 10 patients with spinal cord injury and nine healthy controls. GEO2R analysis was employed to identify genes meeting the criteria of adj. PVal <0.05 and |logFC|>1, which were used to screen differentially expressed genes (DEGs) and assess the expression levels of core target genes (Sadaqat et al., 2023).

### Molecular docking

The structure of curcumin was obtained from the PubChem database and converted from the SDF to Protein Data Bank (PDB) format using OpenBabel 3.1.1 software. The structure of the core target was retrieved from the RCSB PDB database (https://www.rcsb.org) (Kouranov et al., 2006). Using AutoDockTools 1.5.6 software, curcumin and core targets underwent semi-flexible docking; the appropriate box center coordinates and sizes were set, with the number of docking runs set to 50. PyMOL 22.0 software was employed to visualize and generate output for the docking results.

### Molecular dynamics simulations

Two pairs of molecular target complexes with significant research implications and the lowest molecular docking binding energies were simulated and analyzed. GROMACS 2020.6_GPU software was employed to conduct molecular dynamics simulations of the active molecular target complexes, with visualization carried out using QtGrace v026. The TIP3P water model was used for constructing the water model, and protein models tumor necrosis factor (TNF; 6OOY) and matrix metalloproteinase-9 (MMP9; 4HMA) were obtained from the RCSB PDB database, while the composite model was derived from AutoDock Vina docking results. Topology files for proteins and water, along with force field parameters, were generated using commands such as GMX pdb2gmx for topology generation, GMX edition for box building, and GMX solvate for water filling. Ligand molecule topology files and force field parameters were generated and adapted using the LigParGen platform (https://zarbi.chem.yale.edu/ligpargen/) (Dodda et al., 2017). Energy minimization was performed using 5,000 steps in each of the steepest descent and conjugate gradient methods. The system underwent heating stages (NVT and NPT) to reach 26.85°C over 50 ps, followed by a 100 ns simulation under NPT conditions at 26.85°C.

### Cell culture, OGD/R model, and drug treatment

The highly differentiated PC12 cells were purchased from Wuhan Prosay Life Science Company. The PC12 cells were cultured in a high-glucose DMEM containing 10% fetal bovine serum, 100 mg/mL streptomycin, and 100 mg/mL penicillin at 37°C with 5% CO_2_, and the culture medium was changed every 2–3 days. PC12 cells in the logarithmic growth phase were taken, and the high-glucose DEME medium was removed. After three rinses with preheated PBS, sugar-free DMEM (without FBS) was added, and the cells were placed in an anaerobic incubator (1% O_2_, 94% N_2_, and 5% CO_2_) for 6 h. Then, the medium was replaced with a normal medium, and the cells were cultured under normoxic conditions.

Curcumin was purchased from MedChemExpress (98.16% purity). According to the results of the cell counting kit-8 (CCK8) assay, the optimal concentration of curcumin was screened, and 1, 5, 10, 20, 40, and 80 μM concentrations of curcumin were selected for intervention. In the curcumin group, DMEM was used to prepare the curcumin solution.

### Cell counting kit-8 assay

PC12 cells of each group were collected and seeded in 96-well plates at 20,000 cells/well and cultured overnight. According to the proportion of the 1/10 culture solution, CCK 8 working solution was added to each well and cultured in the incubator for 1 h. After that, the absorbance value at 450 nm wavelength was detected using an microplate reader, and cell viability was calculated.

### Western blot analysis

Fresh PC12 cell samples were collected, and proteins were extracted, according to the instructions of the protein extraction kit (Solarbio, Beijing, China). The BCA assay kit (Solarbio, Beijing, China) was used to determine the protein sample. The obtained protein sample (40 μg) was mixed with 5× loading buffer and boiled for 5 min to denature the protein. Then, gel electrophoresis was performed, and the protein was transferred onto a PVDF membrane; next, the membrane was blocked with 5% skim milk at room temperature for 1 h. MMP9 (1:1000, 10375-2-AP; Proteintech, Wuhan, China), TNF-α (1:1000, 17590-1-AP; Proteintech, Wuhan, China), and β-actin (1:5000; Proteintech, Wuhan, China) antibodies were added at 4°C for 24 h. The membrane was washed with TBST three times for 5 min each time and incubated with the secondary antibody at room temperature for 1 h. The membrane was washed with TBST three times for 5 min each time, and the bands were visualized using the ECL chemiluminescence reagent (Thermo Fisher Scientific, United States). The ChemiDoc XRS system (Bio-Rad, United States) was used to expose and detect the bands. ImageJ software was used to calculate the gray value of the bands, and the protein expression level is represented by the gray value ratio.

## Results

### Potential targets of curcumin against spinal cord injury

**FIGURE 2:**
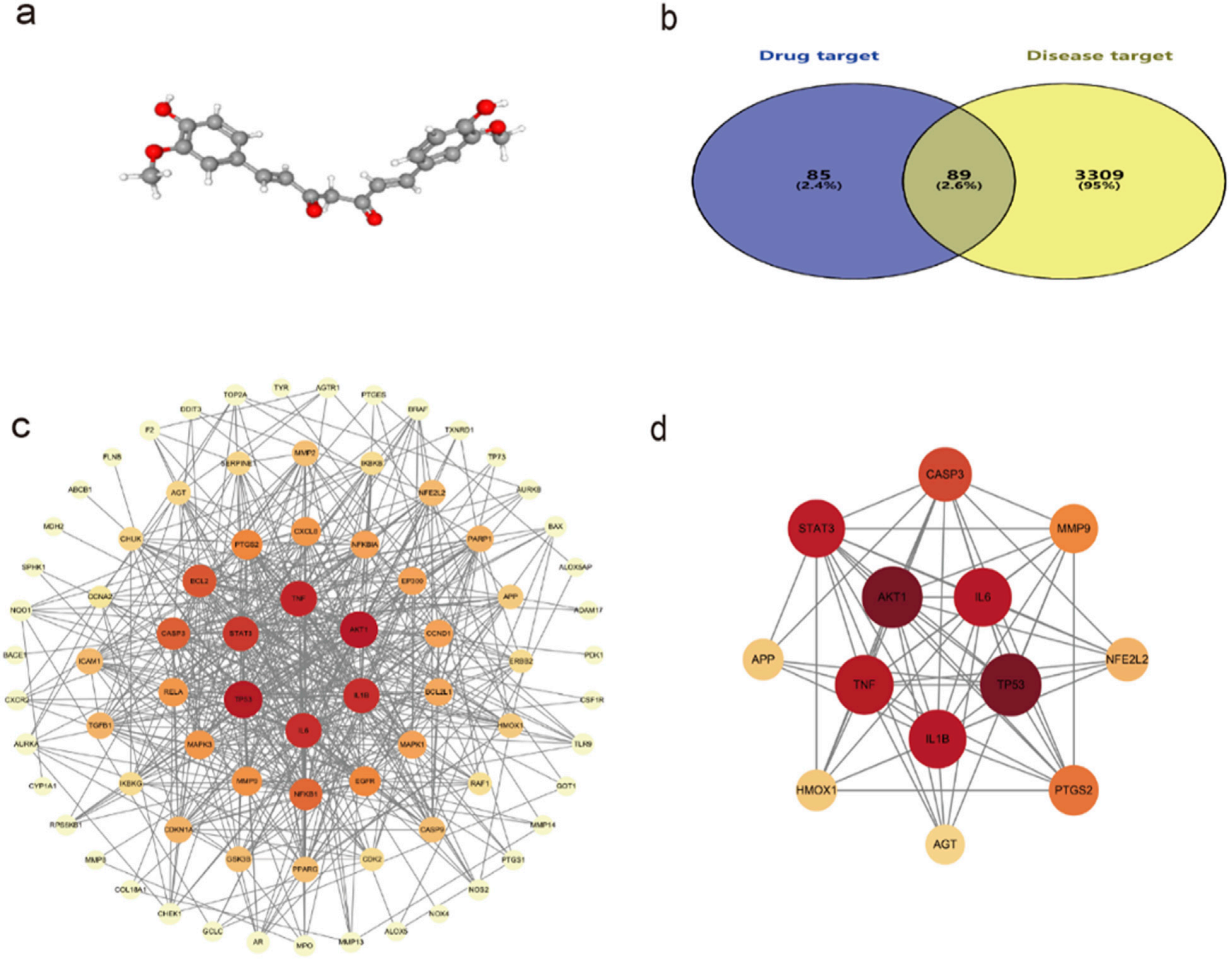
**(a)** 3D structure of curcumin; **(b)** Venn diagram of the common targets of curcumin and SCI; **(c)** Interaction network between curcumin and common targets of spinal cord injury; **(d)** Diagram of the core target interaction network. The darker the color, the larger the sphere, which indicates that the target is more important.

The 3D structure of curcumin was obtained from the PubChem platform (Figure 2A). Curcumin targets were identified through screening in the SwissTargetPrediction, PharmMapper, and CTD databases, resulting in 174 unique targets after eliminating duplicates. Disease-related targets for spinal cord injury were collected from GeneCards, CTD, and OMIM databases, yielding 3,398 unique targets after removing duplicates. An intersection analysis of these two target sets revealed 89 potential targets for curcumin in treating spinal cord injury (Figure 2B).

### Protein interaction network analysis and core target selection

The potential targets of curcumin against spinal cord injury were imported into the STRING platform for constructing a protein–protein interaction network. The results included 89 nodes and 265 edges, analyzed and visualized using Cytoscape 3.10.0 software (Figure 2C). In PPI networks, degree value, closeness centrality, and betweenness centrality represent the number of directly connected nodes of a node, the reciprocal of the average shortest path between a node and other nodes, and the number of shortest paths passing through a node, respectively. When degree value >10, closeness centrality >0.4, and betweenness centrality >0.03, the target was considered an important target (Sun et al., 2023). According to the standard screening targets, the curcumin core may target 13 key proteins related to spinal cord injury, namely, tumor protein p53 (TP53), serine/threonine-protein kinase 1 (AKT1), tumor necrosis factor (TNF), interleukin-1β (Il-1β), interleukin-6 (IL-6), signal transducer and activator of transcription 3 (STAT3), caspase 3 (CASP 3), prostaglandin-endoperoxide synthase 2 (PTGS2), matrix metalloproteinase-9 (MMP9), nuclear factor erythroid 2-related factor 2 (NFE2L2), heme oxygenase 1 (HMOX1), amyloid precursor protein (APP), and angiotensinogen (AGT) (Figure 2D; Table 1).

**TABLE 1:** Core targets of curcumin for spinal cord injury: gene names, degree value, cellular component (CC), and betweenness centrality (BC).

Gene name	Degree value	Closeness centrality	Betweenness centrality
TP53	43	0.65	0.126972
AKT1	42	0.644628	0.094003
TNF	39	0.629032	0.086206
IL-1β	38	0.624	0.097958
IL-6	38	0.614173	0.067243
STAT3	37	0.619048	0.072946
CASP3	32	0.586466	0.039978
PTGS2	27	0.565217	0.056555
MMP9	25	0.537931	0.031224
NFE2L2	18	0.52349	0.062816
HMOX1	15	0.503226	0.04845
APP	15	0.493671	0.038421
AGT	13	0.472727	0.031358

### GO enrichment and KEGG pathway enrichment analyses

**FIGURE 3:**
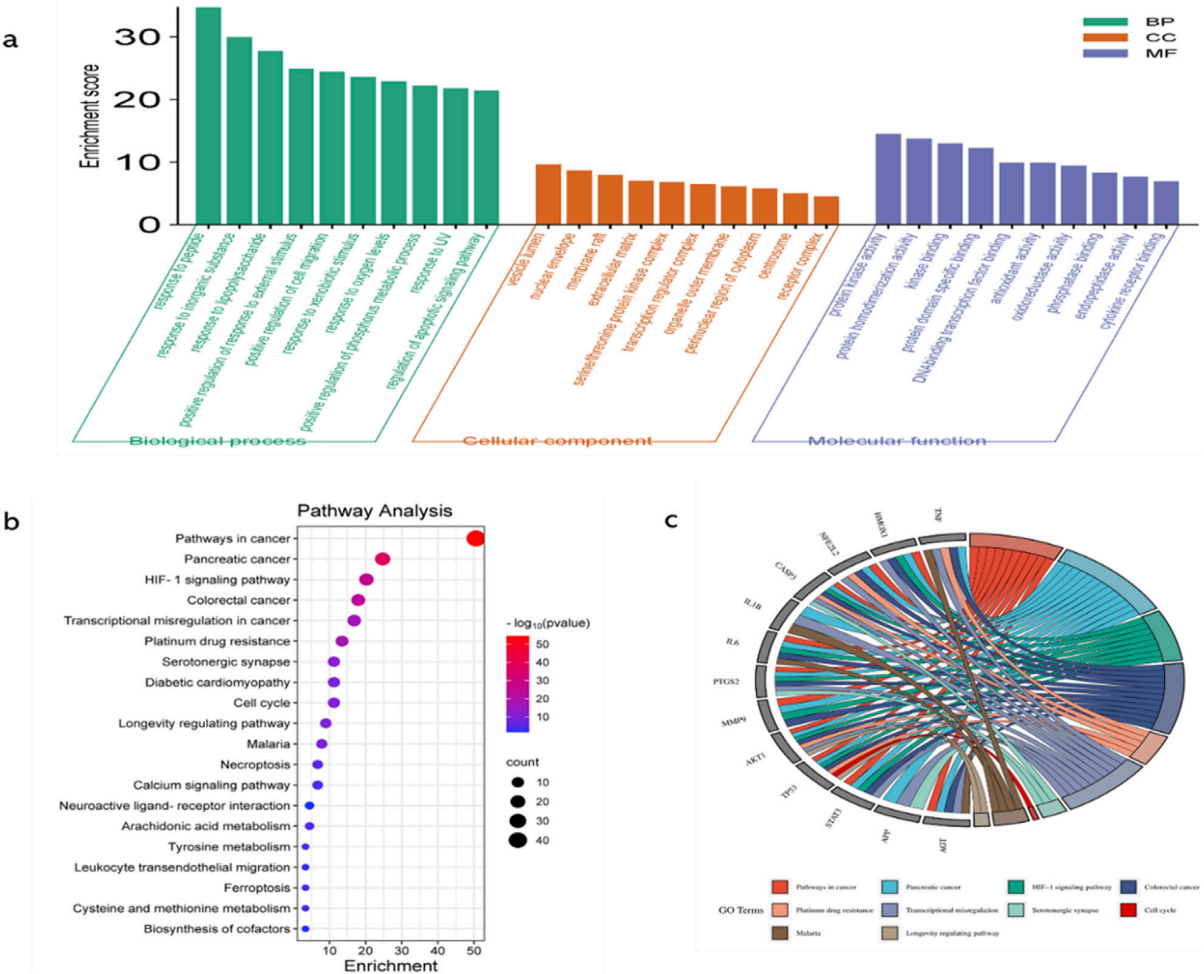
GO functional enrichment analysis, KEGG pathway enrichment analysis, and key target enrichment pathway analysis. **(a)**. GO functional enrichment analysis categorized the targets into three domains, namely, BPs, CCs, and MFs. This analysis provided insights into the biological processes, cellular locations, and molecular activities associated with the targets; higher enrichment scores indicate greater correlation; **(b)** KEGG pathway enrichment analysis (histogram) was conducted to identify significantly enriched pathways. The results are presented as a histogram, where the color gradient (from red to purple) indicates decreasing −log(p) values, and the size of the spheres reflects the enrichment level. Larger spheres represent higher enrichment; **(c)** Key target enrichment pathway analysis was conducted to visualize the connections between targets and pathways using ribbons. The thickness or color intensity of the ribbons indicates the strength of the association, with higher connectivity suggesting greater enrichment of the target in the pathway.

GO function enrichment analysis of potential targets of curcumin against spinal cord injury revealed 237 BPs, 81 CCs, and 93 MFs, and the top 10 were selected according to the p-value and count value for visual analysis (Figure 3A). The top 10 BPs identified through GO enrichment analysis are response to peptide, response to inorganic substance, response to lipopolysaccharide, positive regulation of response to external stimulus, positive regulation of cell migration, response to xenobiotic stimulus, response to oxygen levels, positive regulation of phosphorus metabolic process, response to UV, and regulation of the apoptotic signaling pathway. The top 10 cellular components identified in the GO enrichment analysis are the vesicle lumen, nuclear envelope, membrane raft, extracellular matrix, serine/threonine protein kinase complex, transcription regulator complex, organelle outer membrane, perinuclear region of cytoplasm, centrosome, and receptor complex. The top 10 molecular functions enriched according to GO analysis are protein kinase activity, protein homodimerization activity, kinase binding, protein domain-specific binding, DNA-binding transcription factor binding, antioxidant activity, oxidoreductase activity, phosphatase binding, endopeptidase activity, and cytokine receptor binding.

KEGG pathway enrichment analysis identified 177 pathways, which are primarily associated with hypoxia regulation, metabolism, programmed cell death, inflammation, and other processes. Notable pathways include the hypoxia-inducible factor-1 (HIF-1) signaling pathway, serotonergic synapse, cell cycle, necroptosis, arachidonic acid metabolism, tyrosine metabolism, ferroptosis, and leukocyte transendothelial migration (Figures 3B, C).

#### The “drug–target–pathway–disease” network

**FIGURE 4:**
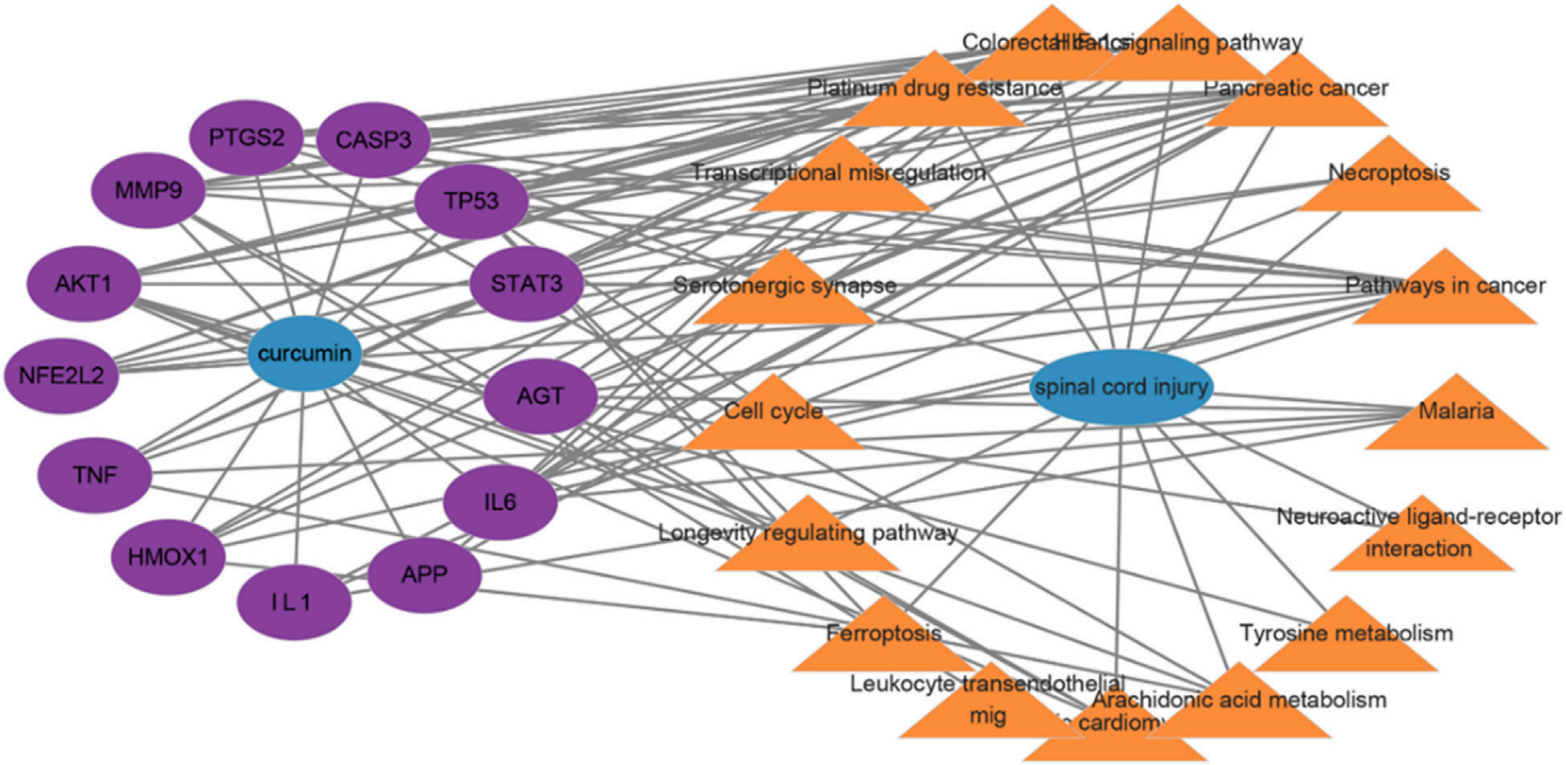
Drug–target–pathway–disease network; lines display the connections between drug targets and disease pathways.

Cytoscape 3.10.0 software was used to construct the drug–target–pathway–disease network. The network diagram revealed that these core targets were closely associated with the top 20 enriched KEGG pathways (Figure 4). Targets of NFE2L2 and HMOX1 exhibit significant antioxidant effects and play a crucial role in ferroptosis pathways (Wang et al., 2023; Eddie-Amadi et al., 2022). CASP3 is involved in the regulation of the apoptosis pathway (Kuo et al., 2019). Among TNF, IL-1, IL-6, MMP9, and STAT3, the latter two play a critical role in the inflammatory response (Tiegs and Horst, 2022; Mantovani et al., 2019; Zhang et al., 2020).

### Microarray data analysis

**FIGURE 5:**
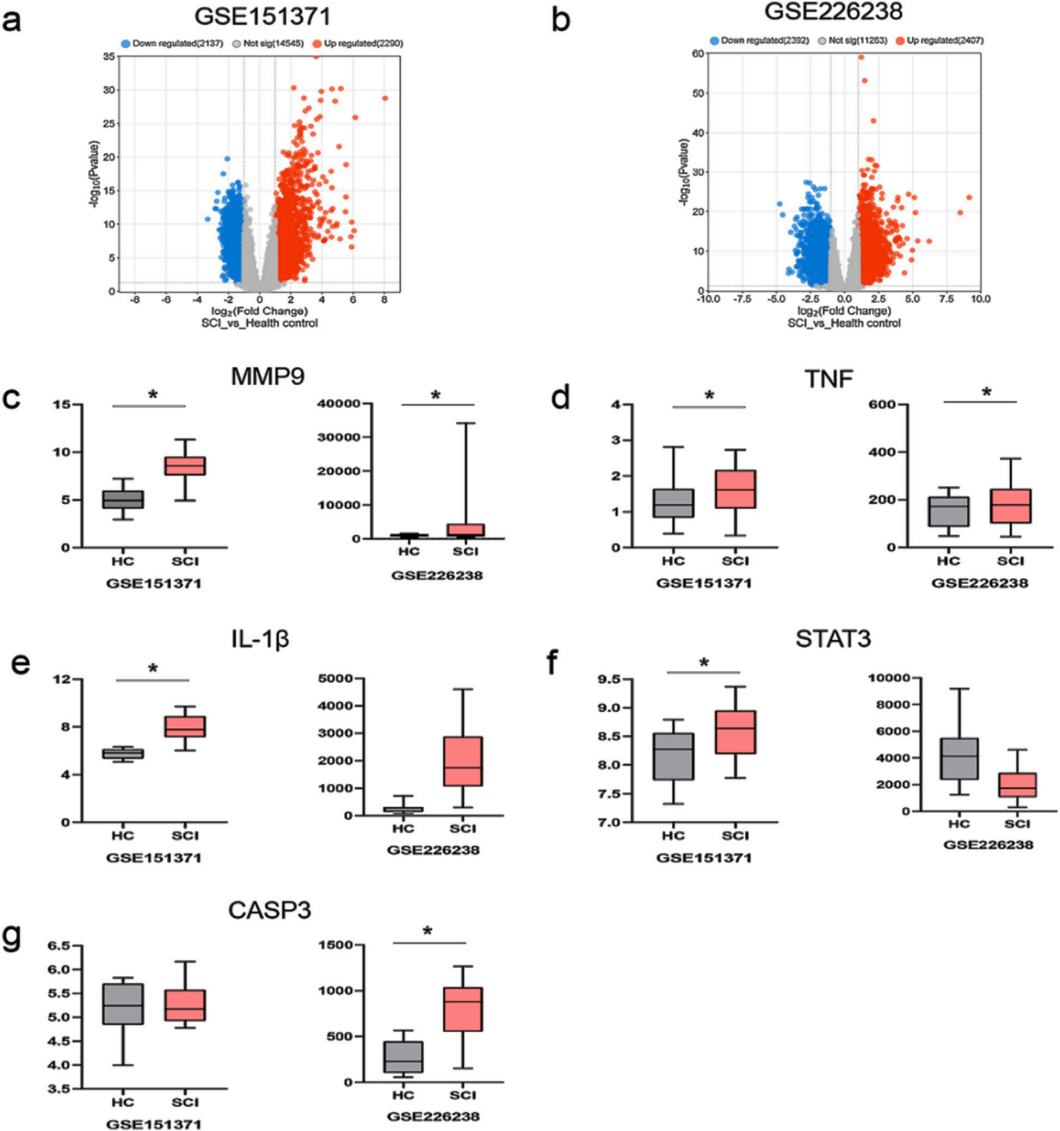
Differentially expressed genes in patients with spinal cord injury and the expression levels of the five most significantly upregulated and downregulated DE-FRGs; **(a)** differentially expressed genes in the dataset GSE151371; red indicates upregulated gene expression, blue indicates downregulated gene expression, and gray indicates no significant difference in gene expression; **(b)** differentially expressed genes in the dataset GSE226238; **(c)** the expression level of MMP9; HC represents healthy control patients, and SCI represents patients with spinal cord injury; **(d)** the expression level of TNF; **(e)** the expression level of IL-1β; **(f)** the expression level of STAT3; **(g)** the expression level of CASP3; *p < 0.05.

Adj. p val <0.05 and |log(FC)| ≥ 1 were used as criteria to screen and analyze differentially expressed genes in patients with an early stage of SCI. In the GSE151371 dataset, 2,290 genes were identified as upregulated, while 2,137 genes were downregulated (Figure 5A). In the GSE226238 dataset, 2,407 upregulated genes and 2,392 downregulated genes were detected (Figure 5B). Among the 13 core targets, MMP9 and TNF gene expressions were upregulated in both the GSE151371 and GSE226238 datasets (Figure 5C, D). Gene expressions of IL-1β and STAT3 were upregulated in the GSE151371 dataset (Figure 5E, F). CASP3 expression was upregulated in the GSE226238 dataset (Figure 5G). MMP9, TNF, IL-1β, STAT3, and CASP3 are potentially crucial in mediating the therapeutic effects of curcumin on spinal cord injury. Molecular docking was employed to further investigate the binding affinities between the drugs and target proteins.

#### Molecular docking

**FIGURE 6:**
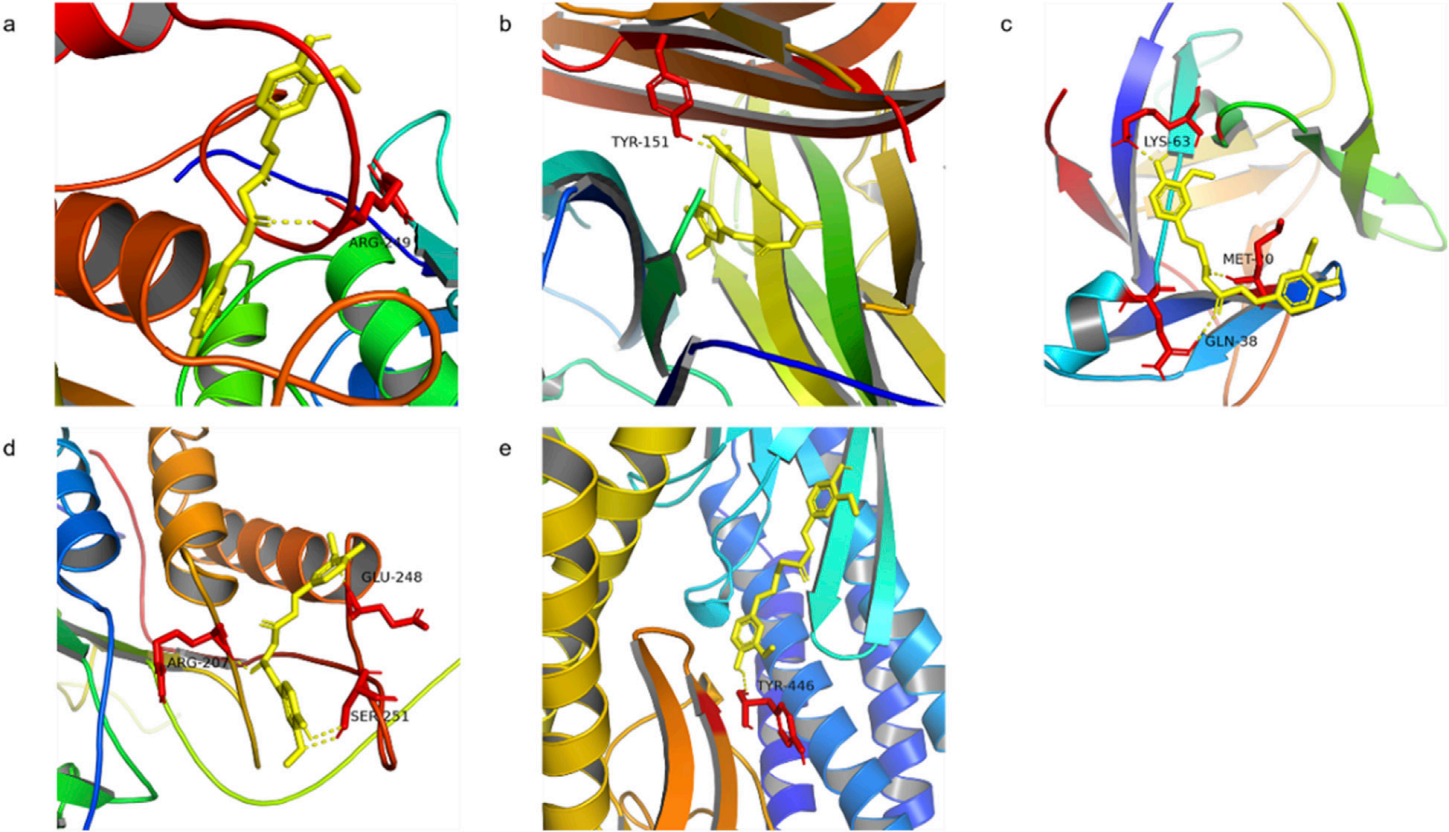
Molecular docking of curcumin with target **(a)** MMP9 docking with curcumin, **(b)** TNF docking with curcumin, **(c)** IL-1β docking with curcumin, **(d)** CASP3 docking with curcumin, and **(e)** STAT3 docking with curcumin.

MMP9, TNF, IL-1β, CASP3, and STAT3 were selected for molecular docking studies with curcumin, and the interactions between these targets and curcumin were analyzed. The structures of the target proteins MMP9 (4HMA), TNF (6OOY), IL-1β (4I1B), CASP3 (3JKF), and STAT3 (6TLC) were retrieved from the RCSB PDB. The 3D conformation of curcumin was obtained by searching for “curcumin” in the PubChem database, and semi-flexible molecular docking was performed using AutoDockTools-1.5.6 software. A lower molecular docking binding energy indicates a stronger interaction between the drug and its target protein. Generally, a binding energy of < -5 kcal/mol suggests that the drug compound is well-docked to the target protein and that the molecular conformation is stable. The results demonstrated that the binding energies of curcumin with all five targets were below −5 kcal/mol, suggesting that these targets interact effectively with curcumin (Figure 6; Table 2). Among them, MMP9 and TNF had the best docking binding ability with curcumin, with binding energies of −8.76 kcal/mol and −8.29 kcal/mol, respectively. MMP9 and TNF may represent key targets of curcumin in the treatment of spinal cord injury, and further investigations were conducted using molecular dynamics simulations.

**TABLE 2:** Protein target molecular docking results of curcumin.

Compound	Target	PDB ID	Residue involved in H bonding	Binding energy (kcal/mol)
Curcumin	MMP9	4HMA	ARG-249	−8.76
Curcumin	TNF	6OOY	TYR-151	−8.29
Curcumin	IL-1β	4I1B	LYS-63; MET-20; GLN-38	−6.06
Curcumin	CASP3	3JKF	ARG-207; GLU-248; SER-251	−5.67
Curcumin	STAT3	6TLC	TYR-446	−5.48

### Molecular dynamics simulation of curcumin and target proteins

The two most promising docking target proteins, along with curcumin, were selected for molecular dynamics simulations. Molecular dynamics simulations over 100 ns were conducted to analyze the binding of the target proteins with curcumin, during which the root mean square deviation (RMSD), root mean square fluctuation (RMSF), and radius of gyration (RG) were computed. The RMSD measures the deviation of the structure conformation from the initial state over time and reflects the stability of the simulation system. The RMSF predicts the average volatility of specific amino acid residues’ positions over time and assesses the flexibility of amino acid residues throughout the simulation process. The RG indicates the compactness of proteins, which, in turn, reflects their stability and co-structures during simulation.

**FIGURE 7:**
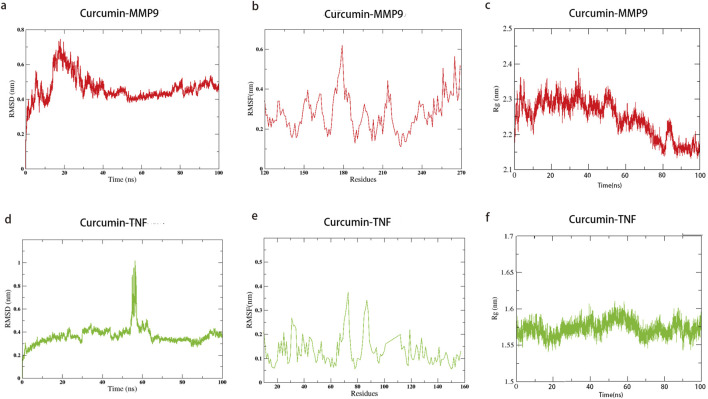
Molecular dynamics simulation of curcumin with MMP9 and TNF; the lines indicate the stability of the binding process of curcumin to the target. Time represents the simulation time, and residue represents the number of molecules corresponding to the molecular dynamics simulation. **(a)** RMSD of curcumin–MMP9; **(b)** RMSF of curcumin–MMP9; **(c)** radius of gyration with curcumin–MMP9; **(d)** RMSD of curcumin–TNF; **(e)** RMSF of curcumin–TNF; **(f)** radius of gyration with curcumin–TNF.

During the molecular dynamics simulation of MMP9 binding to curcumin, the RMSD value showed minimal fluctuation and achieved stability after 40 ns, with RMSD fluctuations of less than 0.1 nm and the lowest RMSF value observed between residues 220 and 225 (Figures 7A, C).This suggests that this region functions as a binding pocket and that curcumin maintains a stable interaction with MMP9. To verify the stability of the connection between MMP9 and curcumin, we calculated the RG values during the simulation. The results showed that the RG value decreased continuously with the extension of the simulation time, indicating that the closeness of the connection between curcumin and MMP9 increased continuously and finally reached a stable state (up and down fluctuated less than 0.1 nm). In the molecular dynamics simulation of TNF binding with curcumin, the average RMSD was low; however, notable fluctuations were observed between 53 ns and 60 ns, indicating potential instability. The RG value also showed fluctuations during this period, which suggested that the stable binding process between curcumin and TNF was broken. The system reached a stable state after 65 ns, with RMSD fluctuations reduced to less than 0.1 nm. The RMSF values reached a minimum between residues 75 and 80 (Figures 7B, D). Combining the results from molecular docking and molecular dynamics simulations, curcumin exhibited a lower binding energy with MMP9 (8.76 kcal/mol) than with TNF. MMP9 and curcumin also reached a stable binding state earlier, exhibiting less volatility. These findings suggest that curcumin may exert its therapeutic effects more effectively through its interaction with MMP9.

### Study on the therapeutic effect and mechanism of curcumin *in vitro* cell experiment

**FIGURE 8:**
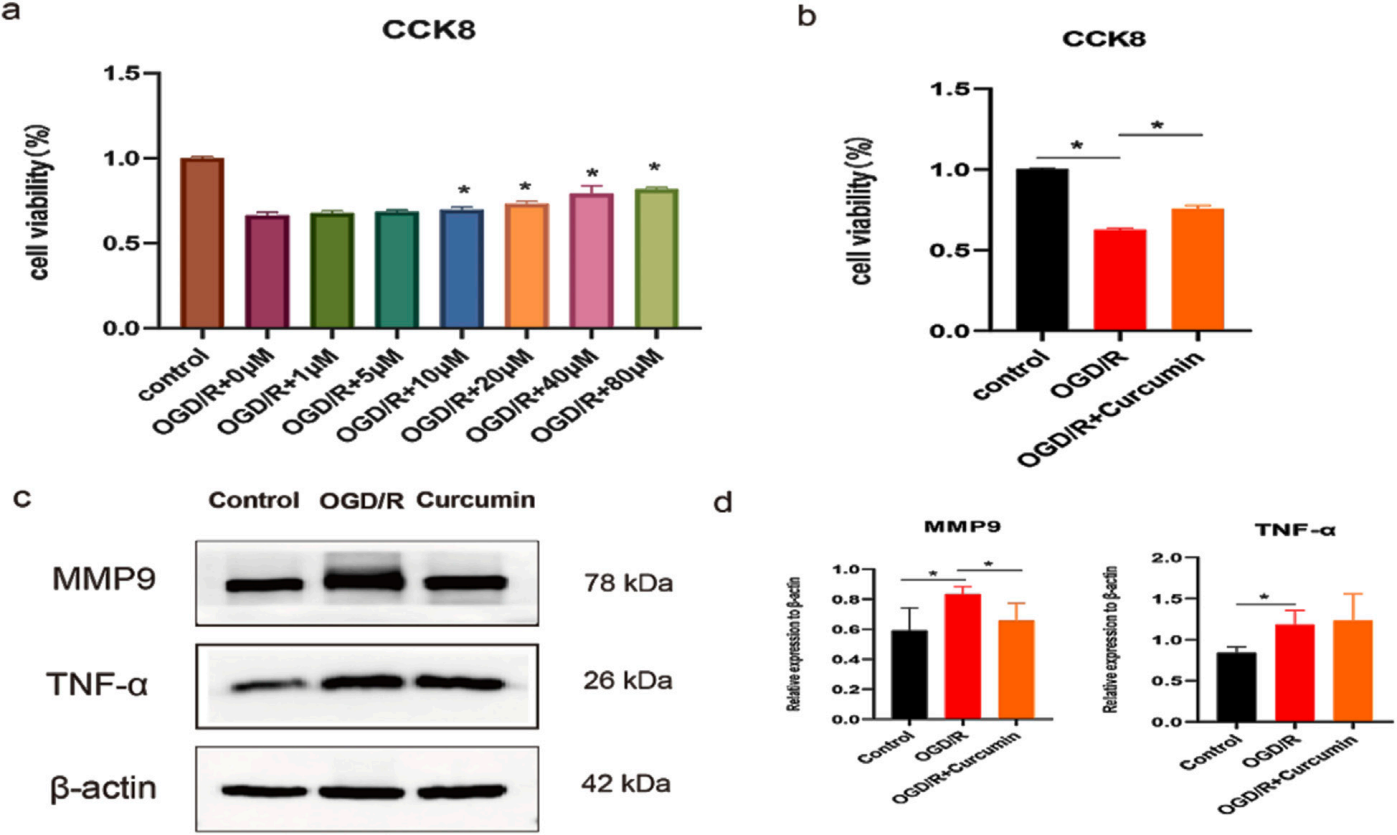
Study on the therapeutic effect and mechanism of curcumin *in vitro* cell experiment. **(a)** Cell activity under different curcumin concentrations; **(b)** cell viability in different groups; **(c, d)** Western blot analysis of MMP9 and TNF-α expression in PC12 cells stimulated with OGD/R; “*” indicates statistical significance; *p < 0.05*.

Molecular docking and molecular dynamics simulations showed that curcumin had good binding stability and activity with proteins MMP9 and TNF, which may be the key targets of curcumin in the treatment of spinal cord injury. Therefore, we constructed an OGD/R model of PC12 cells to simulate spinal cord injury and further studied the therapeutic effect and mechanism of curcumin. First, we treated the cells with different concentrations of curcumin after modeling. CCK8 results showed that the cell activity was increased after the intervention of 10, 20, 40, and 80 μM (*p < 0.05*), and the cell activity showed an increasing trend with the increase in concentration (Figure 8A). We selected 80 μM, the concentration with the highest cell activity, as the experimental concentration in the subsequent implementation. The results showed that curcumin intervention significantly increased cell viability compared with the OGD/R group (Figure 8B), which showed a good therapeutic effect. As a member of the matrix metalloenzyme family, the MMP9 protein is involved in the pathological process of a variety of nervous system diseases (Tokito and Jougasaki, 2016). Studies have shown that MMP9 induces inflammatory responses by disrupting the blood–spinal cord barrier and activating microglia (Mondal et al., 2020). Western blot results showed that compared with the normal control group, the OGD/R group had a significant increase in the protein expression of MMP9 (*P < 0.05*), which was inhibited by curcumin intervention (*P < 0.05*). Zhao et al. (2022) found that the inhibition of MMP9-mediated blood–spinal cord barrier disruption and immune cell infiltration could ameliorate spinal cord injury. TNF family proteins play an important role in inflammation (Siegmund and Wajant, 2023; Jiang et al., 2021), and we performed Western blot analysis on representative TNF-α. The results showed that compared with the control group, the expression of TNF-α was significantly increased in the OGD/R group, while curcumin intervention had no significant effect on the protein expression. Therefore, curcumin may play a therapeutic role by inhibiting the expression of MMP9 protein in SCI.

## Discussion

Spinal cord injuries are associated with high morbidity and mortality rates, and current treatment options are limited. Predominantly, corticosteroids such as prednisone are used, but their clinical benefits, optimal administration period, and severe side effects remain subjects of debate (Sterner and Sterner, 2022). The search for new, effective treatments is of significant research importance. Curcumin has demonstrated considerable potential in treating spinal cord injuries; however, the specific targets and mechanisms of its action remain unclear. Pharmacological and bioinformatics methodologies, including network pharmacology, microarray data analysis, molecular docking, and molecular dynamics simulations, facilitate drug discovery, screening, application, and clinical translation. This study employs network pharmacology and bioinformatics methods to predict the potential targets and mechanisms of curcumin for treating spinal cord injuries. Molecular docking and molecular dynamics simulations are used to investigate how curcumin interacts with these targets and its efficacy, providing a new perspective and a theoretical foundation for advancing curcumin-based treatments for spinal cord injury.

In this study, we first identified the common targets of curcumin and SCI and subsequently screened for core targets using Cytoscape software. Following the methodology established by Sun et al. (2023), targets were considered significant if they met the following criteria: degree value >10, closeness centrality >0.4, and betweenness centrality >0.03. Applying these thresholds, we initially identified 13 potential targets. To validate the relevance of these targets in SCI, we conducted a microarray analysis using the GSE151371 and GSE226238 datasets from SCI patients. DEGs were screened based on the criteria of an adjusted p-value (Adj. p val) < 0.05 and an absolute log fold change (|log(FC)|) ≥ 1 (Sadaqat et al., 2023).The analysis revealed that MMP9, TNF, IL-1β, STAT3, and CASP3 were significantly differentially expressed in early-stage SCI patients, suggesting their critical roles in SCI pathogenesis. Notably, MMP9 and TNF were consistently upregulated in both datasets, indicating their potential as key regulatory targets in SCI. Molecular docking studies further demonstrated the binding stability of curcumin to these core targets (Jiao Yanqi et al., 2023), with MMP9 and TNF exhibiting the most favorable binding energies. This suggests that curcumin can stably interact with these proteins, potentially mediating its therapeutic effects. Additionally, molecular dynamics simulations confirmed the stable binding of curcumin to MMP9 and TNF, supporting the reliability of the docking results. To further validate these computational findings, we conducted cell experiments using curcumin-treated PC12 cells and analyzed the expression levels of MMP9 and TNF. The experimental results aligned with the computational predictions, reinforcing the hypothesis that MMP9 and TNF are key targets through which curcumin exerts its therapeutic effects on SCI.

Our study reveals that curcumin exerts its effects on spinal cord injury through multiple targets and pathways. After filtering, network pharmacology found that 89 potential targets of curcumin against SCI, and further screening narrowed them down to 13 core targets for the treatment of spinal cord injury. We analyzed the differential expression of genes associated with human spinal cord injury. Our analysis identified the differential expression of five core target genes, namely, MMP9, TNF, IL-1β, CASP3, and STAT3, in datasets GSE226238 and GSE151371, suggesting that curcumin targets these genes as a key therapeutic strategy for spinal cord injury. MMP9 is a member of the matrix metalloproteinase family, primarily responsible for degrading extracellular matrix proteins and other bioactive molecules (Mondal et al., 2020). MMP9 plays a critical role in the pathological progression of various nervous system disorders, including the disruption of the blood–brain barrier, promotion of neuroinflammation, and activation of microglia (Tokito and Jougasaki, 2016). Studies have shown that MMP9 contributes to the damage of the blood–spinal cord barrier following spinal cord injury, facilitating immune cell infiltration. The inhibition of MMP9 activation can mitigate pathological damage and promote recovery of motor functions (Jing et al., 2020). The proteins TNF-α and IL-1β, encoded by TNF and IL-1β, respectively, are common pro-inflammatory factors involved in the neuroinflammatory response to spinal cord injury and mediate M1 polarization of microglia (Lin et al., 2021; Freria et al., 2020). Apoptosis represents a critical pathological process in secondary spinal cord injury, with CASP3 serving as a key target in apoptotic pathways. The inhibition of CASP3 can alleviate the pathological damage associated with spinal cord injury (He et al., 2023). STAT3, an important transcription factor, is involved in various pathological processes related to spinal cord injury, including apoptosis (Liu et al., 2023), neuroinflammation (Jiang et al., 2022), and neuronal death (Xiao et al., 2024). Modulating STAT3 expression is, therefore, of significant importance.

The molecular docking binding energies of curcumin with MMP9, TNF, IL-1β, CASP3, and STAT3 were all below −5 kcal/mol, indicating a strong binding affinity between curcumin and these target proteins, with MMP9 and TNF demonstrating the highest affinity for curcumin. Further analysis through molecular dynamics simulations of curcumin interacting with MMP9 and TNF provided insights into the binding process. The binding of curcumin to MMP9 reached a steady state at 40 ns, with the lowest RMSF value observed between residues 220 and 225, indicating a potential binding site in this region. Curcumin binding to TNF reached a steady state after 65 ns, with minimum RMSF values observed between residues 75 and 80. Compared to MMP9, the stability of the curcumin–TNF complex was lower, suggesting that curcumin might more effectively interact with MMP9 to enhance its therapeutic potential for spinal cord injury. We verified the key targets of curcumin intervention by simulating spinal cord injury in PC12 cells and confirmed that MMP9 is one of the key targets of curcumin in the treatment of spinal cord injury. PC12 cells, which are derived from a rat pheochromocytoma, are commonly used to study neuronal differentiation, neurotoxicity, and SCI mechanisms (Li et al., 2023c). However, their use as an SCI model has limitations due to differences from primary neuronal and glial cells. Unlike primary neurons, PC12 cells originate from an adrenal medulla tumor. Although they can differentiate into neuron-like cells with nerve growth factor (NGF) treatment, they lack the full complexity and functionality of primary neurons (Tominami et al., 2024). PC12 cells do not model glial cells, which play critical roles in inflammation, scar formation, and remyelination after SCI. This limits their ability to fully capture SCI pathophysiology. To address these limitations, findings should be validated in primary neuronal and glial cultures to ensure physiological relevance and translational potential.

GO functional enrichment analysis revealed that the potential targets of curcumin in the treatment of spinal cord injury are involved in peptide response, positive regulation of cell migration, apoptotic signaling pathways, extracellular matrix dynamics, transcription regulation, protein kinase activity, antioxidant activity, oxidoreductase activity, and cytokine receptor binding. KEGG pathway enrichment analysis identified 177 signaling pathways, including the HIF-1 signaling pathway, cell cycle regulation, necroptosis, arachidonic acid metabolism, tyrosine metabolism, ferroptosis, and leukocyte transendothelial migration. The HIF-1 signaling pathway, metabolic regulation, ferroptosis, and immune cell regulation are all associated with the pathological processes of spinal cord injury. MMP9, TNF, IL1β, CASP3, and STAT3 are implicated in these pathological processes. As a classical signaling pathway, the HIF-1 signaling pathway is involved in various biological processes, including the regulation of mitophagy (Fu et al., 2020), metabolic reprogramming (Infantino et al., 2021), hypoxia adaptation (Fantis et al., 2023), and glycolysis (Kierans and Taylor, 2021). The HIF-1 signaling pathway regulates neuroinflammatory responses (Li et al., 2020), promotes neuronal repair (Ha et al., 2020) and angiogenesis (Tang et al., 2020), and modulates ferroptosis (Dong et al., 2023). It plays a crucial role in mitigating secondary damage and enhancing functional recovery following spinal cord injury. Curcumin regulates the HIF-1 signaling pathway to promote the recovery of various diseases, including osteoarthritis (Zhang et al., 2021) and fibrosis diseases (Zhang Z. J. et al., 2023). Curcumin can reduce inflammation and oxidative stress mainly by inhibiting the activation of the HIF-1 pathway, which may play a role in spinal cord injury (Bahrami et al., 2018).

Curcumin is well-known for its potent antioxidant properties, similar to those of diarylheptanoids (Sudarshan et al., 2024). These compounds can scavenge reactive oxygen species (ROS) and upregulate endogenous antioxidant enzymes through the Nrf2 pathway, which plays a critical role in mitigating oxidative stress-related damage (Li et al., 2023a). This mechanism is particularly relevant to our study as oxidative stress is a key factor in the pathophysiology of SCI. Recent studies have highlighted the anticancer potential of curcumin, including its ability to inhibit cancer cell proliferation, induce apoptosis, and suppress tumor angiogenesis (Sudarshan et al., 2018). These effects are mediated through the modulation of multiple signaling pathways, such as NF-κB and MAPKs. Although our study focuses on SCI, the broader implications of curcumin’s anticancer activity underscore its multifaceted therapeutic potential. Curcumin has demonstrated significant neuroprotective effects in various models of neurological disorders, including Alzheimer’s disease, Parkinson’s disease, and SCI (Nebrisi, 2021). These effects are attributed to its anti-inflammatory, antioxidant, and anti-apoptotic properties, which align with the mechanisms explored in our study.

Notably, curcumin is involved in a wide range of complex interactions across various signaling pathways and cellular processes. This includes the regulation of the NF-κB signaling pathway, which plays a critical role in inflammation and apoptosis (Tan et al., 2022). However, the broad spectrum of curcumin’s actions may lead to off-target effects, particularly due to its low bioavailability, high concentration effects, and dose-dependent characteristics (Maghool et al., 2023). These factors can result in disruptions to intracellular homeostasis, metabolic imbalances, and even DNA damage (Liu et al., 2022). Such off-target interactions may limit its specificity and therapeutic window. The extensive actions of curcumin could influence the modulation of SCI processes such as neuroinflammation and oxidative stress. Off-target interactions might lead to metabolic disorders or toxicity, complicating recovery from SCI. To address these challenges, nanoparticle delivery systems can be used in future studies to improve targeting accuracy and reduce systemic exposure (Hassanizadeh et al., 2023). In addition, the dose regimen was optimized, and structural modifications were explored to improve selectivity and reduce off-target effects.

In summary, this study systematically employed network analysis, bioinformatics, molecular docking, molecular dynamics simulation, and other techniques to comprehensively investigate the potential mechanisms by which curcumin may aid in the treatment of spinal cord injury. It successfully identified valuable potential targets and provided insights that could enhance the utilization rate and therapeutic efficacy of curcumin. Although computer-based network analysis has yielded significant initial insights into the pharmacological targets associated with curcumin, it is important to acknowledge its inherent limitations. This analytical approach often results in many-to-many correlations that may not correspond to specific pharmacological effects. Although computational methods are beneficial for generating hypotheses, they cannot replace empirical validation. To address these challenges effectively, future research should incorporate rigorous experimental validation to differentiate meaningful pharmacological effects from speculative predictions. This will ensure a more reliable assessment of the therapeutic potential of curcumin for SCI.

## Conclusion

In conclusion, this study employs network pharmacology, microarray analysis, molecular docking, and molecular dynamics simulations to elucidate how curcumin affects spinal cord injury through multiple targets and pathways. Specifically, it reveals the mechanisms by which curcumin inhibits inflammation, apoptosis, and ferroptosis while promoting neuronal restoration. We have pinpointed MMP9 as the target through computer studies; MMP9 exhibits the highest binding affinity and stability with curcumin, making it a potential key target for curcumin’s pharmacological effects. The HIF-1 signaling pathway may play a crucial role in mediating these pharmacological effects. This study provides a theoretical foundation for the clinical application of curcumin in spinal cord injury and underscores the need for further in-depth experimental research.

## Data Availability

The original contributions presented in the study are included in the article/Supplementary Material; further inquiries can be directed to the corresponding author.

## Abbreviations

SCI, spinal cord injury; CTD, Comparative Toxicogenomics Database; OMIM, Online Mendelian Inheritance in Man; GO, Gene Ontology; KEGG, Kyoto Encyclopedia of Genes and Genomes; GEO, Gene Expression Omnibus; MMP9, matrix metalloproteinase-9; TNF, tumor necrosis factor; IL-1β, interleukin-1β; STAT3, signal transducer and activator of transcription 3; CASP3, caspase 3; HIF-1, hypoxia inducible factor-1.
