# Correction: Exploring the therapeutic mechanism of curcumin in spinal cord injury treatment based on network pharmacology, molecular dynamics simulation, and experimental validation

**DOI:** 10.3389/fchem.2025.1636136

**Published:** 2025-06-09

**Authors:** Yongzhi He, Jiachun Lu, Yushan Luo, Rizhao Pang, Xiaoming Hu, Lijuan Ding, Hua Xiao, Yunyun Wang, Wenchun Wang

**Affiliations:** ^1^ School of Clinical Medicine, North Sichuan Medical College, Nanchong, Sichuan, China; ^2^ Department of Rehabilitation Medicine, General Hospital of Western Theater Command, Chengdu, Sichuan, China; ^3^ Department of Rehabilitation Medicine, Xichong County People’s Hospital, Nanchong, Sichuan, China; ^4^ Chengdu Eighth People’s Hospital (Geriatric Hospital of Chengdu Medical College), Chengdu, Sichuan, China; ^5^ College of Medicine, Southwest Jiaotong University, Chengdu, Sichuan, China; ^6^ The Second Affiliated Hospital of Guangzhou University of Chinese Medicine (Guangdong Provincial Hospital of Chinese Medicine), Guangzhou, Guangdong, China

**Keywords:** spinal cord injury, curcumin, natural products of plant origin, molecular mechanism, network pharmacology, molecular dynamics simulation, computer simulation

In the published article, there was an error regarding the **Affiliation(s)** for author Wenchun Wang. Instead of having affiliations 1 and 2, they should only have affiliation 2. The correct affiliations appear above.

The authors apologize for this error and state that this does not change the scientific conclusions of the article in any way. The original article has been updated.

