# Supplementary figures and images for: Exploring the therapeutic mechanism of curcumin in spinal cord injury treatment based on network pharmacology, molecular dynamics simulation, and experimental validation
Source: Front Chem. 2025 Mar 28;13:1568551. doi: 10.3389/fchem.2025.1568551 (PMC11985754; doi:10.3389/fchem.2025.1568551)

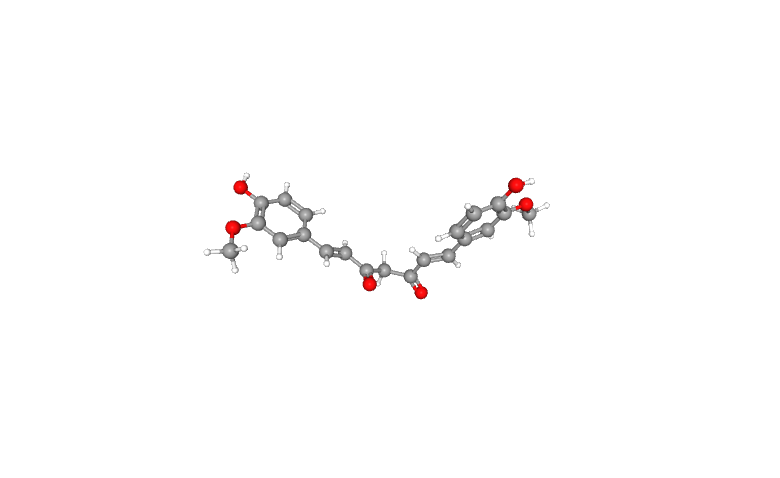

Supplement: Supplementary file 1 [file DataSheet1.zip › Curcumin_Conformer3D_large.png]

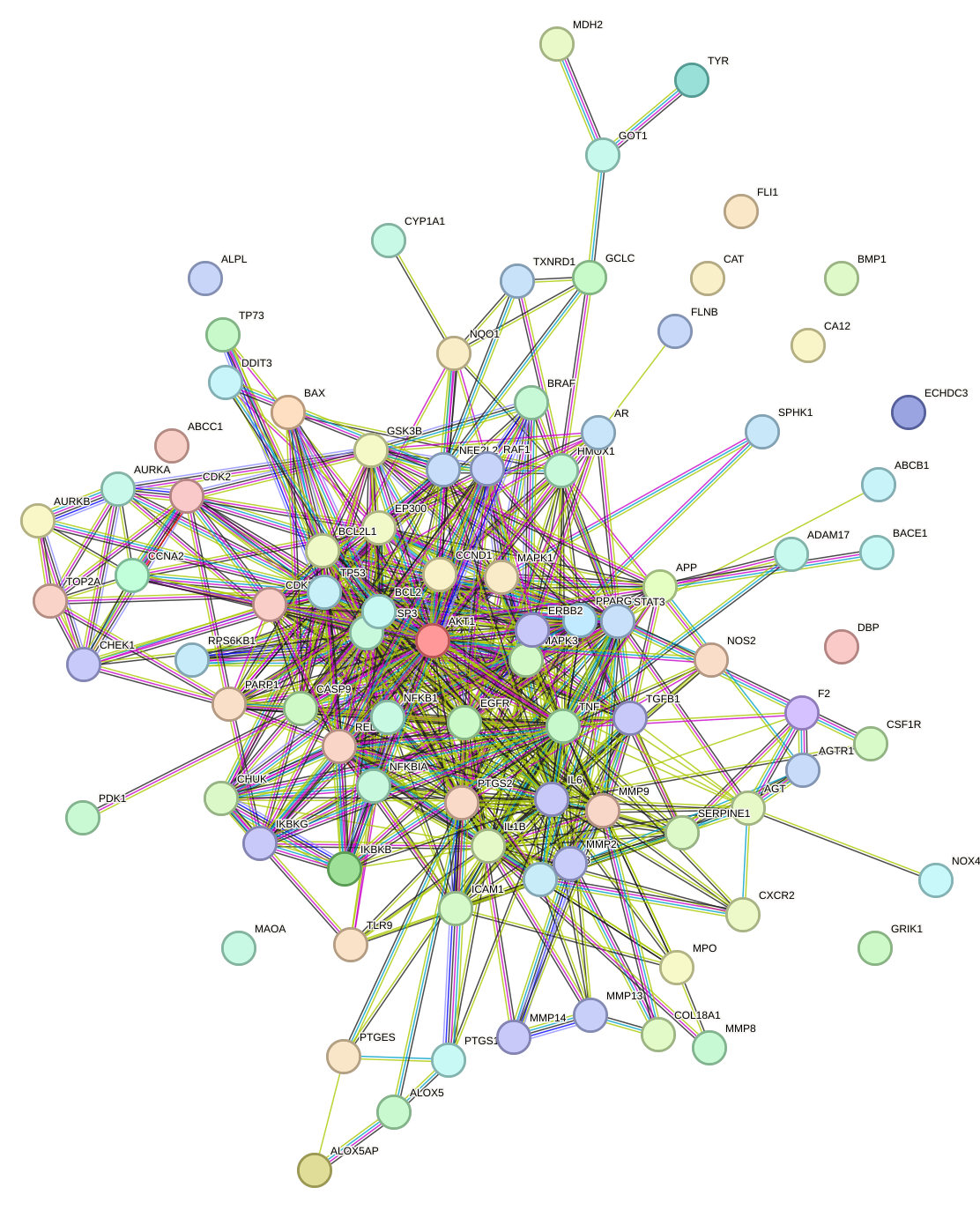

Supplement: Supplementary file 1 [file DataSheet1.zip › string_normal_image.png]

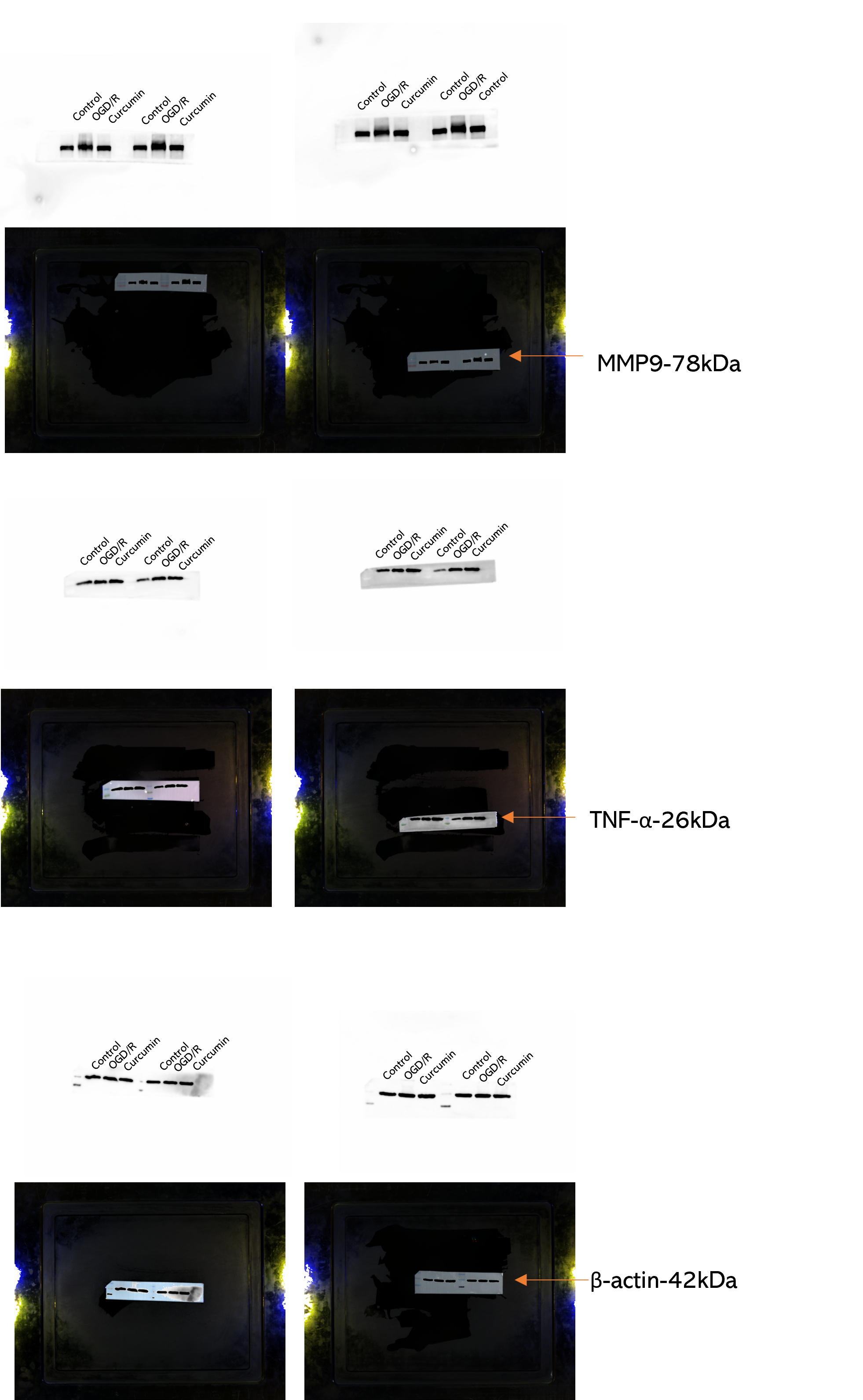

Supplement: Supplementary file 1 [file DataSheet1.zip › western blot.tif]
